# Prevalence and associated risk factors for men being paid for sex in Ethiopia: a multilevel analysis of 2016 Ethiopian demographic health survey data

**DOI:** 10.1038/s41598-024-66748-w

**Published:** 2024-07-09

**Authors:** Ambachew Misanew, Behailu Dessalegn, Zemachu Ashuro

**Affiliations:** 1 Department of Statistics, College of Natural and Computational Science, Hawassa University, Hawassa, Ethiopia; 2 Department of Statistics, College of Natural and Computational Science, Dilla University, Dilla, Ethiopia; 3 School of Public Health, College of Health Science and Medicine, Dilla University, Dilla, Ethiopia

**Keywords:** Prevalence, Risk factors, Men paying for sex, HIV, Multilevel models, Ethiopia, Risk factors, Health occupations

## Abstract

Paying for sex is considered a high-risk sexual behavior, especially among men. Men who pay for sex are perceived to be a bridge group for sexually transmitted illnesses. In sub-Saharan Africa, the prevalence of paid sex among men is approximately 4.3%. Men paid for sex are not studied in Ethiopia. Therefore, the objective of this study was to identify factors associated with men paying for sex in Ethiopia. We analyzed data from the 2016 Ethiopian Demographic Health Survey. In the analysis, 9070 men were included. To identify factors associated with paid-for sex among men, we used a multilevel logistic regression model. A p value less than 0.05 was considered to indicate statistical significance at the 95% confidence interval (CI). In this study, 509 (5.6%) men were ever paid for sex. Men who paid for sex were significantly more likely to be rich [Adjusted Odds Ratio (AOR) = 1.70; 95% CI 1.287, 2.246], widowed or separated (AOR = 1.97; 95% CI 1.142, 3.396), had more sexual partners [AOR = 1.03; 95% CI 1.005, 1.063], had ever been tested for human immunodeficiency virus (HIV) (AOR = 1.50; 95% CI 1.173, 1.916), drank alcohol (AOR = 4.15; 95% CI 3.086, 5.576), and chewing khat (AOR = 2.28; 95% CI 1.822, 2.85); men who had ever paid for sex were significantly less likely to have higher education (AOR = .63; 95% CI .438, .898) and the lowest age at first sex (AOR = .90; 95% CI .870, .924). In conclusion, educational level, wealth status, province, marital status, age at first sexual intercourse, number of sexual partners, HIV status, alcohol consumption status, and chewing khat were significantly associated with men’s paid-for sex. From a public and sexual health perspective, more education is needed for illiterate, widowed, separated, and rich men. Additionally, preventive measures should be taken against men’s behavior through the use of alcohol or khat, having many sexual partners, and having young men.

## Introduction

Paid sex, or the exchange of money, favors, or gifts for sexual intercourse, is a recognized risk factor for HIV infection in developing countries, including among female sex workers (FSWs)^1^. While HIV prevalence varies across regions, some areas within developing countries show surprisingly low rates, even among high-risk populations^2^. Conversely, specific locations such as Zimbabwe, Kenya, Malawi, Côte d'Ivoire, Ethiopia, Tanzania, Benin, and Mali have documented alarmingly high HIV infection rates among FSWs, exceeding 40% in several instances^1^.

Unprotected commercial sex has demonstrably contributed to numerous HIV epidemics worldwide^3^. This inquiry investigates the multifaceted evolution of the sex industry, considering the interplay of socioeconomic factors such as poverty and inequality, ideological shifts such as the proliferation of sexual imagery in advertising, the expansion of the adult entertainment industry in both virtual and physical realms, and technological advancements such as the internet facilitating sex worker advertisement^3^. These changes have simultaneously reduced traditional forms of commercial sex while creating new avenues for women and adolescents to engage in less formal paid sex, potentially altering HIV transmission and acquisition risks^4^.

This study focuses on the association between paid sex and men's risk-taking behaviors in Ethiopia. Paid for sex, inherently linked to power imbalances and potentially involving multiple partners, is known to increase the likelihood of contracting HIV and other sexually transmitted infections (STIs)^5^.

Furthermore, violence against women, encompassing both intimate partner violence and nonintimate partner violence, is a significant public health concern, with over 44% of women in sub-Saharan Africa experiencing at least one form of violence^6^. While the legal and moral implications surrounding sex work remain internationally contested, there is widespread agreement on the necessity of combatting forced sex work^7^.

Despite ratifying the Ministerial Commitment on Comprehensive Sexuality Education (CSE) and Sexual and Reproductive Health Services (SRHS) for adolescents and young people, Ethiopia faces challenges in its implementation^8^. The country also has a high prevalence of STIs, with regional variations documented in the 2016 Ethiopian Demographic and Health Survey (EDHS)^5^. Additionally, the emergence of sex worker organizations advocating for their rights and improved working conditions highlights the ongoing social complexities surrounding sex work in Ethiopia^8^, and Ethiopia faces a significant STI burden, with approximately 4% of the population reporting symptoms in the past year^9^.

While previous studies in Ethiopia have explored factors associated with early sexual initiation and sex with unmarried partners, research on the factors influencing men who pay for sex remains limited^5,10^. Existing studies often employ single-level logistic regression, neglecting the potential influence of regional variations in prevalence^11^. In this study we used a multilevel logistic regression model, to analyze nationally representative data from the Ethiopian Demographic and Health Survey (EDHS), and identified sociodemographic and risk-taking behaviors associated with men who pay for sex in Ethiopia. This approach permits a more sophisticated comprehension of the phenomenon while taking into consideration possible regional differences in factors that contribute to risk.

## Materials and methods

### Study design, setting and period

In this study, we analyzed secondary data from the 2016 EDHS, which were collected from nine regional states and two administrative cities in Ethiopia. The study design employed in the 2016 EDHS was a cross-sectional survey conducted in Ethiopia's urban and rural areas from January 18, 2016, to June 27, 2016.

### Data collection and sampling procedures

The 2016 EDHS data were collected using standard Demographic and Health Survey (DHS) questionnaires adapted by stakeholders from the Ethiopian government and its partners to reflect specific social and cultural issues in Ethiopia. Qualified and trained professionals collected the data under rigorous supervision. The sampling frame contained information about the enumeration area (EA) location, type of residence (urban or rural), and estimated number of residential households.

The 2016 EDHS sample was stratified and selected in two stages. Each region was stratified into urban and rural areas, yielding 21 sampling strata. EA samples were selected independently from each stratum in two stages. In the first stage, 645 EAs (202 from urban areas and 443 from rural areas) were selected. In the second stage, a fixed number of 28 households were selected per cluster. A total of 12688 men age between 15 and 59 years were eligible for interviews. However, 9070 men were given a response to the question, “Have you ever paid or exchanged money for sex?”.

### Study variables

*The dependent variable* was “Have you ever paid for sex?” Responses were either yes or no and were generated from the EDHS variables, which were denoted as 1 = “yes”, 0 = “no”.

*Independent variables* Based on previous literature and biological knowledge, the independent variables included in the analysis were sociodemographic characteristics (age, educational level, religion, wealth status, employment status, residence status, marital status, and province) and risky sexual behaviors (age at first sexual intercourse, sexual activity, number of sexual partners, chewing khat, alcohol consumption status, HIV status, and knowledge of HIV).

### Data access and methods of statistical analyses

The EDHS 2016 survey data are available on the DHS program website. Descriptive analyses were conducted to describe the demographic characteristics and risky sexual behaviors of the participants using the mean with standard deviation (SD) for continuous variables and the number (%) for categorical variables. The chi-squared test was used to identify association between dependent and independent variables.

Single-level and mixed logistic models were constructed to examine the independent associations between demographic characteristics, risky sexual behavior, and sex. A final model was developed by removing variables with the highest p values, refitting the model, and repeating the step until all p values of the included variables were less than 0.05. Parameter estimates for single-level and multilevel logistic analyses were calculated and are presented as 95% confidence intervals (CIs) and p values. STATA version 15 (Stata Corps LP, Texas, USA) was used for all the data analyses^12^.

### Multilevel logistic model

A multilevel logistic model, also referred to in the literature as a hierarchical model, can account for the lack of independence across nested data levels (e.g., individual households nested within a provincial area). Standard logistic regression assumes that all experimental units (in our case, households/individuals) are independent in the sense that any variable affecting the dependent variable has the same effect in all regions. Multilevel modeling relaxes this assumption and allows the effects of these variables to vary across provinces.

### General two-level logistic regression models

First, we introduce a simple two-level model used to analyze binary data. Let j denote the level-2 units (provinces) and i denote the level-1 units (nested household individuals). Assume that there are j = 1,…, m level-2 units and i = 1,…, n_j_ level-1 units nested within each level-2 unit j. The total number of level-1 observations across level-2 units is given by the $n = \sum\nolimits_{j = 1}^m {n_j}$.

The response variable is denoted by Y_ij_

$${Y}_{ij}=\left\{\begin{array}{ll}1, &\quad {\text{if ith respondent from the jth region is ever paid for sex}}\\ 0, &\quad {\text{if ith respondent from the jth region is not ever paid for sex}}\end{array}\right.$$

### The multilevel random intercept model

Men’s paid sex prevalence is likely to vary in different geographical locations, either due to cultural, religious, or economic differences, and these effects enable us to include these unknown variations in the model using random effects. Furthermore, individuals living in the same region may be more similar to each other than individuals living in other regions, as they share similar cultures and living styles. In addition, there is a correlation between paid sex status and living in the same household. These indices introduce intraclass correlation, which is a measure of the degree of similarity among the paid sex status of members of the same cluster, i.e., region or household. Therefore, this study employed a multilevel logistic regression model with region-specific random effects to account for the intraclass correlation and hence quantify the variation in a paid-sex outcome that is accounted for by the region variances.

In the random intercept model, the intercept is the only random effect, meaning that the groups differ concerning the average value of the response variable; however, the relationship between the explanatory and response variables cannot differ between groups. We assume that there are variables that potentially explain observed success and failure. These variables are denoted by X_*h*_ (h = 1, 2, …, k), with their values indicated by X_*hij*_. Since some or all of these variables could be level one variables, the probability of success is not necessarily the same for all individuals in a given group^13^. Therefore, the probability of success depends on the individual as well as the group and is denoted by P_*ij*_.

The random intercept model expresses log odds, that is, the logit of P_*ij*_*,* as the sum of a linear function of the explanatory variables., i.e.,

1 $$\text{Logit(p}_{ij})=\log \left( {\frac{{{p_{ij}}}}{1 - pij}} \right) = {\beta_{0j}} + {\beta_1}{x_{1ij}} + {\beta_2}{x_{2ij}} + \cdots + {\beta_K}{x_{kij}} = \upbeta_{0j}+\sum\limits_{h = 1}^k {{\beta_h}{x_{hij}}}$$

where the intercept term ${\beta_{0j}}$ is assumed to vary randomly over a region. ${\beta }_{0j}$ is independently and normally distributed with mean zero and variance ${\delta }_{\beta }^{2}$, in short ${\beta }_{0j}\sim N\left(0, {\delta }_{\beta }^{2} \right)$, and is given by the sum of the average intercept β_0_ and group-dependent deviations U_0*j*_, that is,

β_0*j*_ = β_0_  + U_0*j*_, i = 1, 2, ….,n; and j = 1, 2, …., m.

As a result, we have:  

2 $$\text{Log}\left( {\frac{{{p_{ij}}}}{1 - pij}} \right) = \upbeta_{0}+ \sum\limits_{h = 1}^k {\beta_h} {x_{hij}} + U_{0j}$$

Thus, a unit difference between the X_*h*_ values of two individuals in the same group is associated with a difference in β_*h*_ in their log odds, or equivalently, a ratio of exp (β_*h*_) in their log odds. In Eq. (2), β_0_ + $\sum\nolimits_{h = 1}^k {{\beta_h}{X_{hij}}}$ is the fixed part of the model*.* The remaining U_0*j*_ is known as the random part of the model. It is assumed that the residual U_0*j*_ is mutually independent and normally distributed, with a mean of zero and variance ${\delta_0}^2$. When the logistic model is used, the residual at level one is assumed to follow the standard logistic distribution with mean 0 and variance $\frac{{\uppi }^{2}}{3}=3.29$. The intraclass correlation coefficient (ICC) of the within-group variation for dichotomous variables is defined as ICC = $\frac{{\updelta }_{0}^{2}}{{\updelta }_{0}^{2}+3.29}$, where ${\delta_0}^2$ is the variance of the two error terms and 3.29 is the variance of the standard logistic distribution^14^.

### Ethical approval and consent to participate

This study did not require ethical approval or participant permission because it was a secondary data analysis of survey data from the Measure DHS program that was made publicly available. We asked the DHS Program for permission to obtain and utilize the data from http://www.dhsprogram.com for this study, and we were granted this permission. The methods approved by the Institution Review Board for DHS public-use datasets prevent respondents, households, or sample communities from being identified in any way.

## Results

### Sociodemographic characteristics of the study participants

This study included 9070 men, and nearly 509 (5.6%) men had ever been paid for sex. A total of 86 (16.9%) men age between 25 and 29 years reported that having paid or exchanged money for sex accounted for the highest proportion among the other age groups. Only 15 (2.9%) respondents were aged 15–19 years, accounting for the smallest proportion of men who reported ever paying or exchanging money. Compared to respondents who reported not having paid for sex, those who reported having paid for sex were not significantly different within any age group (p = 0.258). The proportions of men living in urban areas who had ever paid for sex and those who had not ever paid for sex (28.8% vs. 55.0%, p < 0.001) were significantly different. The first two highest proportions (29.0% and 27.2%) of men who reported having ever paid or exchanged money for sex lived in Addis Ababa city and the Tigray region, respectively.

A comparison of men who had ever paid to exchange money for sex and men who had not ever paid or exchanged money for sex across provinces (regions) revealed a significant association (p < 001). Most respondents who reported having paid for sex and those who reported not having paid for sex with orthodox religious followers (74.5% and 41.6%, respectively) reported having a primary education (34.4% and 37.0%, respectively).

Regarding wealth status, 46.7% of respondents who reported having paid for sex and 70.7% of respondents who reported not having paid for sex were rich at the economic level. Moreover, over 80.6% of the respondents reported not having paid for sex, and 59.3% reported having paid for sex-reported marriage. In the past, 8.4% of respondents reported not having paid for sex, and 11.4% reported having paid for sex. Men who reported having paid for sex and those who reported not having paid for sex were found to be significantly different in terms of religion, educational level, economic level, marital status, and employment status (Table 1).

**Table 1:** Sociodemographic characteristics of men paying for sex in Ethiopia (n = 9070).

Variable	Category	Men paid or not paid for sex with count (%)		P value
		No paid count (%)	Paid count (%)	
Age in years	15–19	303 (3.5)	15 (2.9)	.258
	20–24	961 (11.2)	57 (11.2)	
	25–29	1613 (18.8)	86 (16.9)	
	30–34	1424 (16.6)	78 (15.3)	
	35–39	1287 (15.0)	66 (13.0)	
	40–44	1138 (13.3)	72 (14.1)	
	45–49	810 (9.5)	55 (10.8)	
	50 and above	1025 (12.0)	80 (15.7)	
Region	Tigray	705 (9.5)	138 (27.2)	.000*
	Afar	565 (7.6)	40 (7.9)	
	Amhara	1151 (15.5)	31 (6.1)	
	Oromia	1160 (15.6)	32 (6.3)	
	Benishangul	756 (10.2)	15 (3.0)	
	SNNPR	1071 (14.4)	13 (2.6)	
	Gambela	634 (8.5)	49 (9.7)	
	Addis Adaba	784 (10.6)	147 (29.0)	
	Dire Dawa	591 (8.0)	42 (8.3)	
Residence	Urban	2467 (28.8)	280 (55.0)	.000*
	Rural	6094 (71.2)	229 (45.0)	
Religion	Orthodox	3561 (41.6)	379 (74.5)	.000*
	Catholic	65 (0.8)	1 (0.2)	
	Protestant	1435 (16.8)	42 (8.3)	
	Muslim	3410 (39.8)	84 (16.5)	
	Other	90 (1.0)	3 (0.6)	
Educational level	No education	2994 (35.0)	114 (22.4)	.000*
	Primary	3168 (37.0)	175 (34.4)	
	Secondary	1218 (14.2)	122 (24.0)	
	Higher	1181 (13.8)	98 (19.3)	
Economic status	Poor	3389 (39.6)	116 (22.8)	.000*
	Middle	1174 (13.7)	33 (6.5)	
	Rich	3998 (46.7)	360 (70.7)	
Marital status	Never in union	1330 (15.5)	167 (32.8)	.000*
	Married/together	6900 (80.6)	302 (59.3)	
	widowed/separated	115 (1.3)	24 (4.7)	
	Divorced	216 (2.5)	16 (3.1)	
Employment status	Unemployed	716 (8.4)	58 (11.4)	.022*
	Employed	7845 (91.6)	451 (88.6)	

P values are from Pearson’s chi-squared test. *Significant variables (p < 0.05).

### Risky sexual behaviors

As shown in Table 2, the average age at first sexual intercourse for men who reported not having paid for sex was slightly greater than that for men who reported having paid for sex (20.54 years ± SD = 4.26 vs. 19.12 years ± SD = 3.32, p value < 0.001). Men who reported not having paid for sex had a significantly lower mean number of sexual partners in the past than did those who reported having paid for sex (mean of 98 ± 1.53 vs. 1.3 ± 4.37, p < 0.001). Moreover, a significantly greater proportion of men who reported not having paid for sex were sexually active in the last four weeks than men who reported having paid for sex (67.7% vs. 58.5%, p < 0.001). Approximately 78% of men who reported having paid for sex and 54.7% of men who reported not having paid for sex had been tested for HIV in the past; 36.6% of men who reported not having paid for sex and 44.4% of men who reported having paid for sex had been chewing khat, 46.2% of men who reported not having paid for sex, and 84.8% of men who reported having paid for sex had been drinking alcohol. A significant association was observed between the risky sexual behavior of men and men who had paid or not paid money for sex. There was no statistically significant difference in men’s knowledge of HIV between men who reported having paid for sex and those who reported not having paid for sex ***(***Table 2***).***

**Table 2:** Association of sex-paid risky sexual behavior among men (n = 9070).

Variable	Category	Men paid or not paid for sex with count (%)		p value
		No paid	Paid	
Age at first sexual intercourse	Mean age at first sex (± SD)	20.54 (4.26)	19.12 (3.32)	.000*
Sex partners	Mean sexual partners(± SD)	.98 (1.53)	1.3 (4.37)	.000*
Sexual activity	Active in last 4 weeks	5800 (67.7)	298 (58.5)	.000*
	Not active in last 4 weeks	2761 (32.3)	211 (41.5)	
HIV test	No	3881 (45.3)	112 (22.0)	.000*
	Yes	4680 (54.7)	397 (78.0)	
Chewing khat	No	5331 (63.4)	278 (55.7)	,001*
	Yes	3084 (36.6)	221 (44.3)	
Drinking alcohols	No	4527 (53.8)	76 (15.2)	.000*
	Yes	3888 (46.2)	423 (84.8)	
knowledge on HIV	No	3384 (40.9%)	211 (42.5)	.505
	Yes	4882 (59.1)	286 (57.5)	

P values are from Pearson’s chi-squared test. *Significant variables (p < 0.05).

### Factors associated with paid-for sex

The focus of this study is on the group (region) level of the hierarchy, and the aim is to show the differences among men paid for sex in regions. Furthermore, the available data have a hierarchical structure: the first-level units are men, and the second-level units are regions. Consequently, we propose different specifications for a multilevel mixed logistic model (Table 3).

**Table 3:** Multilevel mixed-effect null model without explanatory variables.

Fixed part for paying money for sex	Coefficients	S.E	Z value	P value
intercept (cons)	− 2.959	.308	− 9.60	0.000

Random part	Estimate	S.E	Ratio	
Random intercept: Var (cons) = ${\delta_0}^2$	.825	.406	2.03	.000
Intraclass correlation (ICC)	.2005			

*Significant variables (p < 0.05) from the mixed logistic null model, without explanatory variables.

From the model estimates in Table 3, the intercept of men paying money for sex in region j was − 2.959 + U_oj,_ and the estimated variance ${\delta_0}^2$ was 0.825. The ratio of the intercept variance to its standard error is 2.03, indicating that the between-region variance differs from zero. In Table 4, 20.05% of the total variations in men paid for sex were accounted for by level two units (regions), indicating that the multilevel mixed-effect model is appropriate since it considers variation within regions.

**Table 4:** Factors associated with men paying for sex in the multilevel mixed-effects logistic model.

Variable	Category	$\beta$	Std. err	Z	P value	95% CI ($\beta$)	AOR	95% CI(AOR)
Educational level	No education(ref.)							
	Primary	− .109	.142	− 0.77	0.443	− .387, .169	0.897	.679, 1.184
	Secondary	− .098	0.172	− 0.570	0.570	− .435, .240	0.907	.647, 1.271
	Higher	− .467	0.183	− 2.550	0.011*	− .826, − .108	0.627	.438, .898
Wealth status	Poor(ref.)							
	Middle	− 0.004	0.214	− 0.020	0.987	− .422, .415	0.996	0.656, 1.515
	Rich	0.531	0.142	3.730	0.000*	.252, .809	1.700	1.287, 2.246
Marital status	Never in union(ref.)							
	Married/together	− 0.429	0.120	− 3.570	0.000*	− .665, − .193	0.651	.514, 0.824
	widowed/separated	0.678	0.278	2.440	0.015*	.132, 1.223	1.969	1.142, 3.396
	Divorced	− 0.289	0.293	− 0.990	0.324	− .863, .285	0.749	.422, 1.330
Age at first sexual intercourse	Agesex	− 0.109	0.015	− 7.060	0.000*	− .139, − .079	0.897	.870, .924
Num. of sex	num. sex. partner	0.033	0.014	2.320	0.020*	.005, .0609	1.034	1.005, 1.063
Had test HIV	No(ref.)							
	Yes	0.405	0.125	3.230	0.001*	.1596, .651	1.499	1.173, 1.916
Chewing khat	No(ref.)							
	Yes	0.824	0.114	7.220	0.000*	.600, 1.047	2.279	1.822, 2.850
Alcohol drinking	No(ref.)							
	Yes	1.423	0.151	9.430	0.000*	1.127, .719	4.148	3.086, 5.576
Random part	Region	Coef	Std. err					
	Var (cons) = ${\delta_0}^2$	0.601	0.301			.225, 1.606		
	ICC	0.155						

*OR* odds ratio, *CI* confidence interval.

LR test vs. logistic model: chibar2(01) = 190.73, Prob >  = chibar2 = 0.0000, *significant variables (p < 0.05) from the multilevel mixed-effect logistic model.

As indicated in Table 4, considering the random intercept mixed model, all variables had a significant effect on men paid for sex. The ICC value was 0.155, showing that 15.5% of the variation accounted for the dependent variable "paid for sex” at the regional level. Multilevel mixed effect modeling provides a significantly better result than single-level modeling without considering random effects, which can be seen from the results of the LR test (LR test vs. binary logistic regression chi-square = 190.73, P value = 0.000), confirming that the difference is significant at 0.05, which verifies the better goodness of fit for the multilevel mixed logistic model.

According to Table 4, men with higher education levels were 37.3% less likely to be paid for sex than those who had no education. Economically rich men were 70% more likely to pay money for sex than poor men were. Married men were 35% less likely, and widowed/separated men were 96.9% more likely to be paid money for sex than men who were never in unions.

Men with more sexual partners had a greater probability of paying for sex (OR = 1.034, p < 0.001). Men’s age at first sexual encounter had a negative relationship with men who paid for sex (OR = 0.897, p < 0.001). Therefore, men of greater age at first sex were less likely to pay money for sex. Men who had ever been tested for HIV (OR = 1.499, p = 0.001) were 49.9% more likely to pay money for sex than men who had not been tested for HIV. Men who chewed khat and drank alcohol were 2.279 and 4.148 times more likely to pay for sex, respectively, than men who did not chew khat and drink alcohol (Table 4).

## Discussion

In this study, there were significant differences in the proportion of men who paid for sex between the regions and provinces. The highest probability of men paying for sex was observed in Tigray Province, followed by Afar and Addis Ababa. The demographic and socioeconomic characteristics of each region may explain this discrepancy. This study was supported by a study in Cambodia in which the prevalence of selling sex was statistically associated with the province^15^. Significant differences were observed between cities in the proportion of men who reported paying for sex in this interurban study^16^.

In this study, men who had higher education levels were 37.3% less likely to be paid money for sex than men who had no education. A similar study conducted in Spain^2^ supported our study related to education and reported that paying for sex increased with lower educational levels (AOR = 1.8, 95% CI 1.2 to 2.7 in those with less than a secondary education compared to those who had a university education). However, another study in sub-Saharan Africa^17^ reported that the odds of paying for sex were significantly greater among men who had completed only primary (AOR = 1.31) or secondary education (AOR = 1.13) than among those with no formal education. However, men with a tertiary level of education had lower odds of paying for sex than men with no formal education (AOR = 0.77)^17^, and higher levels of education were generally associated with decreased odds of paying for sex^16^. This finding was inconsistent with the results of the present study. This might be because in Ethiopian culture, men who have higher educational levels have great respect, and most literate persons do not participate in clubs and bars; thus, they are less likely to be exposed to paying money for sex.

The other variable that showed a significant association with paying money for sex in this study was wealth status; that is, economically rich men were 1.7 times more likely to pay money for sex than poor men were. Our study concluded that the richest men pay money for sex more than the poorest men do, which is in contrast to the findings of another study performed in sub-Saharan Africa^17^, which reported that men in the richer (AOR = 0.87) and the richest (AOR = 0.83) wealth quintiles had lower odds of paying for sex than men in the poorest wealth quintile and, based on a similar study in rural Malawi^16^, had higher household wealth (as wealth increases, men are less likely to have paid for sex (AOR = 0.828, p < 0.05). It might be that the richest men may be able to pay or exchange any gifts or money for sex because they may be seen as more “popular” and thus may be more likely to be able to have any beautiful females with money; specifically, it can be common in a developing country, such as Ethiopia.

Another study revealed that the odds of paying for sex were the lowest among married men, who were 34.9% less likely than men who were never in unions, and widowed/separated men were 96.9% more likely to pay money for sex than men who were never in unions. The findings of this study were consistent with those of Seidu in sub-Saharan Africa^17^. Men who were divorced (AOR = 4.52), separated (AOR = 3.70), or never married (AOR = 2.58) had greater odds of paying for sex than those who were married. In addition, multivariate analyses showed that married and cohabitating men in São Paulo and Cuernavaca were significantly less likely to have a lifetime history of paying for sex^16,18^. A possible reason for this finding could be the legal nature and greater acceptance of marriage in Ethiopia. Thus, married men are less likely to participate in sexual intercourse because it leads to a breakdown in marriage.

This study also revealed a statistically significant association between age at first sexual encounter and paid money for sex. Age at first sexual intercourse was negatively correlated with having paid money for sex (AOR = 0.897, p < 0.001). Therefore, men who were older at first sexual intercourse were less likely to pay money for sex. In this study, the reported age at first sexual intercourse in men who reported having paid for sex was 19.12 (± 3.32), whereas in other studies, it was 18.8 (± 1.9)^17^, and respondents who reported having paid for sex were slightly younger than respondents who reported not having paid for sex. In addition, greater age at first sexual intercourse is negatively associated with the number of sexual partners last year, such that being older or having a later age of sexual initiation, on average, reduces the number of partners in the past year^18^; paying for sex was strongly associated with early sexual debut^19^. A possible reason for this finding might be that younger individuals may not have experienced and initiated sex initiation at a younger age.

In this study, men with a greater number of sexual partners had a greater probability of paying money for sex and showed a positive association with men paying money for sex. This finding is similar to that of a study in Norway^19^ in which paying for sex was strongly associated with having more sexual partners. Moreover, a positive association has been observed between paying for sex and the number of sexual partners in the past year^18^. Dizechi et al.^15^ confirmed that men reporting more than 4.6 sexual partners (the average number of sexual partners) were significantly more likely to pay for sex. This average was higher than that reported in other studies of men who paid for sex in Cambodia. The reason might be that men may have experienced considerable exposure to risk factors due to having multiple sex partners, and those who pay for sex may continue to pay for sex.

Men who had been tested were 49.9% more likely to pay money for sex than men who had not been tested for HIV. There is also evidence that having paid for sex is associated with having had an HIV test^19^. In contrast, other studies have suggested that HIV testing does not affect the likelihood of men paying for sex^15^.

Finally, in this study, men who chewed khat and drank alcohol were 2.279 and 4.148 times more likely to pay money for sex than men who did not chew khat and drink alcohol, respectively. This finding also agrees with a study^2^ that stated that men who had drunk more than once in the last 30 days (AOR = 1.9, 95% CI 1.3–2.6) were more likely to pay money for sex than men who had never drunk. However, there are no studies related to this finding. Having paid for sex is also associated with the use of injectable drugs or alcohol^19^.

### Limitations of the study

This study has limitations since the EDHS survey relied on self-reports from respondents, which could lead to recall bias because participants were asked about events that occurred at least seven years before the survey. In addition, although these characteristics are significant determinants of men's paid sex, the EDHS did not gather data on community attitudes, norms, values, or beliefs about paid sex.

## Conclusion and recommendations

In this study, having paid for sex was associated with high-risk sexual behavior and represented a public health problem. Paid sex among men in Ethiopia is associated with sociodemographic variables, including education, wealth status, province/region, marital status, and risky sexual behaviors, including age at first sexual intercourse, number of sex partners, HIV testing, alcohol consumption, and chewing khat. In addition, based on the multilevel mixed-effects model, it was observed that men paying money for sex varied across Ethiopia. To help men pay for sex in Ethiopia, programs should seek to improve their educational level, and men should be restricted to a single sexual partner. Rich men also invest their income for purposes other than sexual intercourse. From a public and sexual health perspective, more health awareness education is needed for illiterate, widowed or separated and rich men. Preventive measures should also address men’s behaviors across alcohol or drug usage, marital status, and age. Furthermore, special attention should be given to regional and geographic variations.

## Data Availability

The data used for this study were obtained from the Ethiopian Demographic and Health Surveys (EDHS) and are publicly available at https://dhsprogram.com/data/dataset/Ethiopia_Standard-DHS2016.cfm.

## Abbreviations

AOR: Adjusted odds ratio
CI: Confidence interval
CSE: Comprehensive sexuality education
EDHS: Ethiopian Demographic and Health Survey
EA: Enumeration area
FSWs: Female sex workers
SRHS: Sexual and reproductive health services
WHO: World Health Organization
HIV: Human immune deficiency virus
STIs: Sexually transmitted infections
SD: Standard deviation
LR: Log-likelihood ratio
ICC: Intraclass correlation coefficient
